# Seasonal Dynamics of Chlorophyll Fluorescence in the Evergreen *Peumus boldus* and the Semideciduous *Colliguaja odorifera* Under Field Conditions

**DOI:** 10.3390/plants15020276

**Published:** 2026-01-16

**Authors:** Sergio Espinoza, Marco Yáñez, Eduardo Martínez-Herrera, Carlos Magni

**Affiliations:** 1 Departamento de Ciencias Forestales, Facultad de Ciencias Agrarias y Forestales, Universidad Católica del Maule, Av. San Miguel 3605, Talca 3460000, Chile; espinoza@ucm.cl; 2 College of Forestry, Agriculture, and Natural Resources, University of Arkansas at Monticello, 110 University Ct, Monticello, AR 71656, USA; yanez@uamont.edu; 3 CESAF, Facultad de Ciencias Forestales y de la Conservación de la Naturaleza, Universidad de Chile, Avenida Santa Rosa 11365, La Pintana, Santiago 8003636, Chile; crmagni@uchile.cl

**Keywords:** photoprotective mechanisms, photosynthetic performance, sclerophyllous, winter depression

## Abstract

We used chlorophyll fluorescence techniques to investigate seasonal variations in photosystem II (PSII) quantum yield in five-year-old saplings of the sclerophyllous *Peumus boldus* Molina (evergreen) and *Colliguaja odorifera* Molina (semideciduous) planted in a semiarid site with a Mediterranean-type climate. Chlorophyll fluorescence rise kinetics (OJIP) were monitored monthly for one year (September 2024 to September 2025). With this information, we estimated the relative deviation of the performance index (PI_ABS_) of each species from the average PI_ABS_ in each season (denoted as ∆PI_ABS_). *P*. *boldus* was associated with destruction of PSII reaction centers and incapacity for electron transport, i.e., higher values of parameters ABS/RC (effective antenna size of an active reaction center) and F_0_ (minimal fluorescence), whereas *C*. *odorifera* was associated with higher photosynthetic performance i.e., higher values of PI_ABS_, PI_TOT_ (total performance index), F_V_/F_0_ (ratio between variable and minimal fluorescence), and F_V_/F_M_ (maximum quantum yield of primary PSII photochemistry). P_IABS_ exhibited a 52 and 38% reduction (i.e., −∆PI_ABS_) during spring and winter in *P*. *boldus*, but an increase (i.e., +∆PI_ABS_) of 52 and 37% in the same seasons for *C*. *odorifera*. *P*. *boldus* was considerably more depressed during the winter–spring season than the summer months. This suggests that PSII function in *P*. *boldus* is more sensitive to low temperatures in winter and spring than the lack of water and high temperatures during summer.

## 1. Introduction

The Mediterranean semiarid ecosystems of Central Chile are characterized by dry and warm summers, with high temperatures and radiation loads, and mild winter temperatures. Whereas this ecosystem covers c.a. 2 million hectares between 30° and 40° S [1] and sustains a diverse number of endemic plant species [2], it faces increasing threats due to severe and intense droughts, fires, land use change, and degradation [3]. The evergreen *Peumus boldus* Molina and the semideciduous *Colliguaja odorifera* Molina are two typical species cohabiting in these ecosystems that play an ecological role. *P*. *boldus* is an important source of timber, tannins, and medicinal products [4], whereas *C*. *odorifera* tend to resist drought stress at early outplanting [5] and is therefore a good candidate for initiating ecological restoration programs. However, both species exhibit an array of different adaptive strategies to survive under the harsh conditions found in Mediterranean-type climates [6,7]. The presence of *P*. *boldus* is more abundant on South- and East-facing slopes (i.e., the wettest in the Southern Hemisphere) and in altitudes lower than 1000 m a.s.l. than in other conditions [8,9]. It exhibits leaf rolling as a mechanism to decrease transpiration [10,11], but a low photosynthetic performance at early outplanting during the summer months [5]. In contrast, as drought-deciduous species increase with aridity [12], *C*. *odorifera* appears on dry north-facing slopes (i.e., the driest in the Southern Hemisphere) [8,13]. The species exhibits leaves with inclination angles higher than 65° [5], which allows it to reduce high radiation loads during summer and helps in maintaining photosynthesis [14]. Its semideciduous habit and high leaf shedding in summer [6] also help in balancing water absorption with transpiration demand during the summer and dry months. Contrary to *P*. *boldus*, *C*. *odorifera* seedlings exhibit high performance in photosynthetic parameters related to absorption and trapping of photons, heat dissipation, and electron transport during summer [5].

In addition to coping with the combination of summer stress factors, plants are also prone to suffering winter photoinhibition. During summer, drought, solar radiation, and high temperatures impair photosynthetic performance, but in winter, low temperatures negatively affect plants’ metabolic functions [15], especially in evergreen sclerophyllous species [16], causing damage to physiological and metabolic processes [17]. Despite temperatures in Mediterranean-type ecosystems never being too low or too high to exceed the operational limits of photosynthesis [12], no attempt has been made to understand the photosynthetic responses that *P*. *boldus* and *C*. *odorifera* have developed to cope with and survive throughout the different seasons of the year, particularly during the two critical periods for photosynthetic activity, i.e., during summer drought and low winter temperatures. Both seasons challenge the successful restoration of sclerophyllous species in the context of current and future global change scenarios [18]. The lack of success in the establishment phase causes important failures in restoration projects [19], especially when species are not selected properly. It is thus of paramount importance to select well-adapted species in restoration projects and to implement silvicultural techniques for successful establishment after planting. A preliminary step is to provide insights into seedling photosynthetic performance and inform species-based selection for restoration properly.

The use of chlorophyll fluorescence is a widely employed tool in photosynthesis research as it enables rapid and non-destructive evaluation of the structure and function of the photosynthetic apparatus in response to environmental conditions [20]. This technique provides a quantitative estimate of electron transport rates and photosystem PSII (PSII) efficiency, allowing a rapid evaluation of light trapping, primary photochemistry, and efficiency of electron transport [21]. Typical responses of sclerophyllous species under the harsh conditions of Mediterranean ecosystems are a reduction in the performance index (PI_ABS_), the most important parameter related to energy conservation from photons absorbed by the PSII antenna to the reduction of PSI acceptors [22,23]. In terms of summer temperature, it has been reported that the sclerophyllous *Quercus ilex* L. has an optimal range for photosynthetic performance up to 35 °C in summer [15]. In the same species, it was observed that during summer, the potential efficiency of PSII photochemistry (F_V_/F_M_) and PI_ABS_ decreased to values close to 0.5 and < 5, respectively, in seedlings at full sun, suggesting photoinhibition [24]. In contrast, temperatures below –5 °C in winter can induce ice formation in the mesophyll cells of *Q*. *ilex*, inhibiting CO_2_ uptake [15]. In the sclerophyllous *Nerium oleander* L. it was reported that F_V_/F_M_ drastically fell to values close to 0.5 during winter [14]. Similarly, in *Q*. *ilex*, a decrease in F_V_/F_M_ and PI_ABS_ during the winter months was reported (i.e., F_V_/F_M_ ~ 0.68 and PI_ABS_ ~ 8), clearly indicating inefficiency of the photosynthetic apparatus [24]. In the present study, we aimed to investigate the seasonal dynamics of chlorophyll fluorescence in two common sclerophyllous species cohabiting sites with Mediterranean-type climate after 5 years on a Mediterranean drought-prone site.

## 2. Results

### 2.1. Microclimatic Conditions at the Study Site

**Figure 1:**
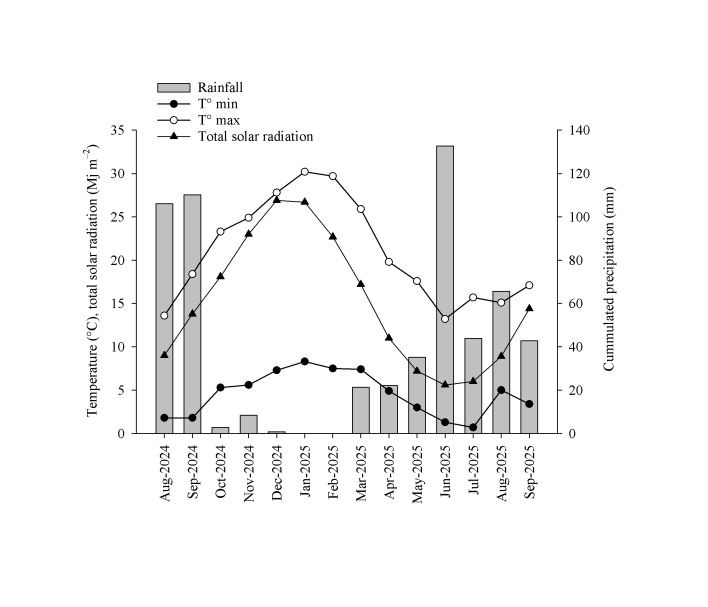
Monthly maximum and minimum temperature (open and solid circles, respectively), total solar radiation (triangles), and cumulated precipitation (bars) at the study site (data obtained from Estancia Flora agroclimatic station (35°29′ S, 72°13′ W), San Javier, Chile).

Precipitation was highest in August and September 2024 and June 2025 (106, 110, and 133 mm, respectively) (Figure 1). Rainfall was low from October to December 2024 and absent in January and February 2025. Air temperature was minimum in July 2025 (0.7 °C), but no temperatures below zero occurred during the study period. The maximum average air temperature was 31 °C in January 2025. Solar radiation was maximum in December 2024 and January–February 2025 and minimum in July 2025.

### 2.2. Relationships Among Variables and Species

**Figure 2:**
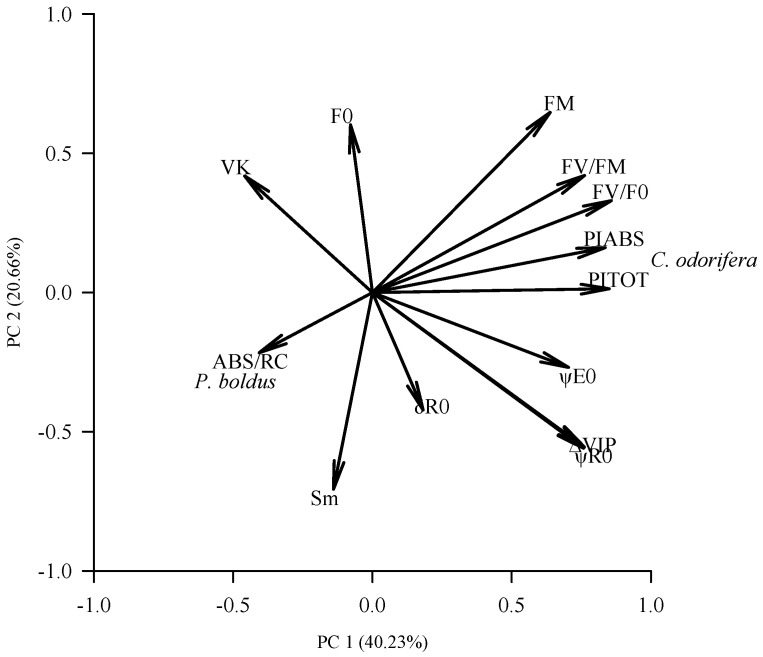
Distribution of the JIP-test parameters in the species surveyed according to the principal component analysis (PCA). F_0_ = minimal fluorescence from a dark-adapted leaf; FM = maximal fluorescence from a dark-adapted leaf; FV/FM = maximum quantum yield of primary PSII photochemistry; FV/F0 = ratio between variable and minimal fluorescence; Sm = normalized area above the curve from F0 to FM; ABS/RC = effective antenna size of an active reaction center (RC); PIABS = performance index for energy conservation from photons absorbed by PSII to the reduction of intersystem electron acceptors; PITOT = performance index for energy conservation from photons absorbed by PSII antenna to the reduction of PSI acceptors; ψE0 = probability that the energy of a trapped excitation is used for electron transport beyond QA; ψR0 = quantum yield for reduction in the end electron acceptors at the PSI acceptor side; δR0 = efficiency with which an electron from the intersystem electron carriers is transferred to reduce end electron acceptors at the PSI acceptor side; VK = relative fluorescence at the K-step; ΔVIP = efficiency with which a PSII trapped electron is transferred to final PSI acceptors.

The principal component analysis indicated that the first (PC 1) and second (PC 2) principal components explained 61% of the variability in the species under study (Figure 2). The PC1 captures the seedling performance index (mainly PI_ABS_ and PI_TOT_), whereas the PC2 captures the number of electron acceptors and the area above the curve (Sm, F_0_, and F_M_). PI_ABS_ and PI_TOT_ appeared to be positively correlated with F_V_/F_M_ and F_V_/F_0_ (correlation > 0.68) but negatively correlated with ABS/RC and V_K_ (–0.43 < r < –0.18). *C*. *odorifera* is associated with higher PI_ABS_ and PI_TOT_, whereas *P*. *boldus* is associated with higher ABS/RC.

### 2.3. Seasonal Variations in Chlorophyll Fluorescence Associated with the Species

There were variations in almost all chlorophyll fluorescence parameters at the species level, except for Sm, F_M_, and V_K_ (Table 1). The highest PI_ABS_, PI_TOT_, and F_V_/F_M_ were found in *C*. *odorifera* (18.9, 19.9, and 0.71, respectively), whereas *P*. *boldus* exhibited the lowest values of these parameters but the highest ABS/RC and F_0_ (3.74 and 168.5, respectively). Seasons were also different in the analyzed parameters. The highest PI_ABS_ and F_M_ were reported in spring (21.1 and 523.6, respectively), and the lowest values during autumn (4.8 and 355.1, respectively). The winter photoinactivation degree (PhI) was 0.35 and 0.17 for *P*. *boldus* and *C*. *odorifera*, respectively.

**Figure 3:**
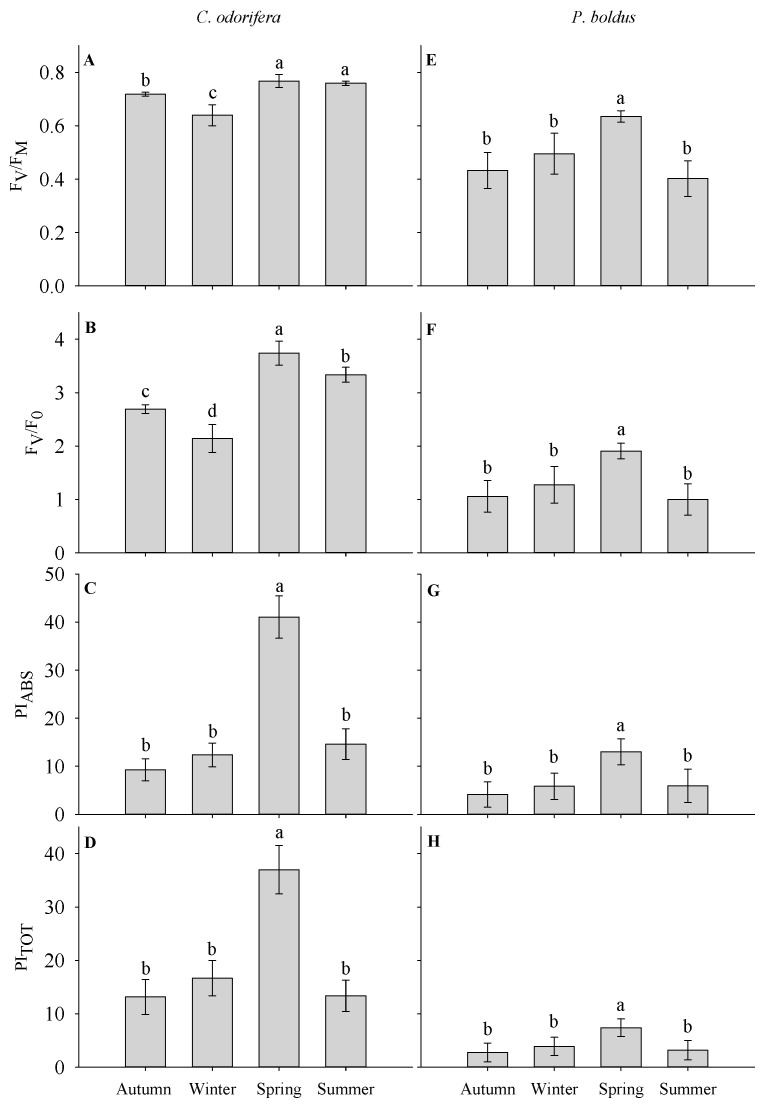
Average values for F_V_/F_M_ (**A**,**E**), F_V_/F_0_ (**B**,**F**), PI_ABS_ (**C**,**G**), and PI_TOT_ (**D**,**H**) for *C*. *odorifera* (panels **A**–**D**) and *P*. *boldus* (panels **E**–**H**) in each season. Lowercase letters indicate differences across seasons. Each bar represents average values for nine seedlings on each date of measurement, and error bars represent the standard error.

**Figure 4:**
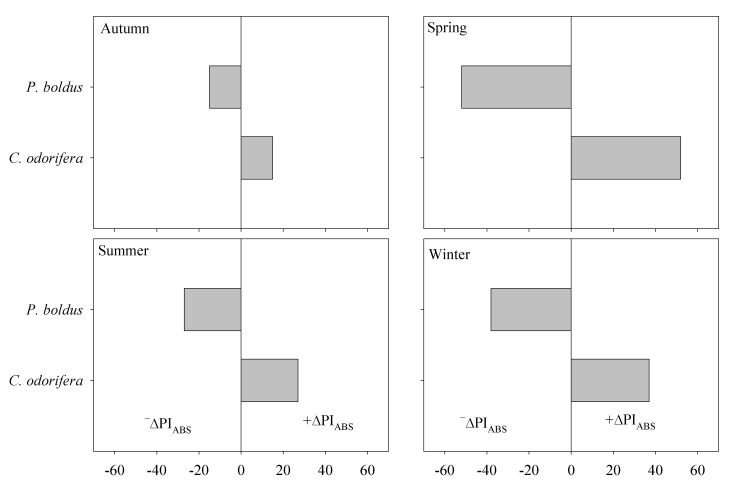
Relative deviation of the performance index (PI_ABS_) of each species in relation to the average PI_ABS_ of all data in each season. The 0 represents the average PI_ABS_ for each season.

Species differed across seasons in variables related to PI_ABS_ (interaction Species × Season in Table 1). Both species exhibited the highest PI_ABS_, PI_TOT_, F_V_/F_0_, and F_V_/F_M_ during spring, but the lowest values differed according to the species (Figure 3). F_V_/F_0_ and F_V_/F_M_ were low in *C*. *odorifera* in autumn, but *P*. *boldus* exhibited low values of all parameters across seasons. Because of this, we estimated the relative deviation of PI_ABS_ (the most important parameter of plant vitality) of each species from the average PI_ABS_ in each season separately. We found that *C*. *odorifera* was the best performer as it has +∆PI_ABS_ of 33% on average across the entire year. In contrast, *P*. *boldus* was a poor performer, as it negatively deviates from PI_ABS_ in all seasons, but during winter and spring, it exhibited the lowest performance (−∆PI_ABS_ = 52 and 38%, respectively) (Figure 4).

## 3. Discussion

Most restoration projects with sclerophyllous species in Central Chile lack of success in the establishment phase because species are planted without knowledge on ecophysiological requirements. In this study we present the first attempt to provide physiologically based information that may guide the selection of appropriate species to restore degraded sites characterized by Mediterranean-type climates in Central Chile. We report on the photosynthetic performance between *P*. *boldus* and *C*. *odorifera*, which was markedly different throughout the year. Both species cohabit the same ecosystem, but their dominance and abundance are more niche-specific. It is well established that *C*. *odorifera* replaces *P*. *boldus* as aridity increases, being thus more common on north-facing slopes (i.e., higher temperatures and irradiation), whereas *P*. *boldus* is more abundant on South and East-facing slopes [8,13]. Adaptations to these different habitat conditions seem to have influenced the seasonal fluctuations in chlorophyll fluorescence in the common garden site and worked better in *C*. *odorifera* than in *P*. *boldus*. Although *P*. *boldus* is evergreen with a long photosynthetic period, its seasonal performance was lower than the semideciduous *C*. *odorifera* with short periods of activity but superior photochemical efficiency.

During summer, *C*. *odorifera* had F_V_/F_M_ of 0.76 and a positive deviation of PI_ABS_, indicating a physiological state relatively close to optimal [25]. In *P*. *boldus* F_V_/F_M_ reached minimum values of 0.40, and PI_ABS_ negatively deviated from the reference PI_ABS_, indicating a depression of the photosynthetic apparatus [14,26]. It might be possible that this is a strategy of *P*. *boldus* aimed at increasing the non-radiative dissipation of excitation energy, but this hypothesis needs to be further elucidated. The decrease in all photosynthetic parameters in *P*. *boldus* during summer suggests structural damage to the PSII and inefficient thermal dissipation of energy. This was evidenced by the lower values of F_V_/F_0_ (i.e., the efficiency of the water-splitting system), which is considered a proxy for heat dissipation [27]. In contrast, *C*. *odorifera* exhibited higher values of F_V_/F_0_. These results suggest that in summer, *C*. *odorifera* has superior capacity to maintain higher PSII center openness during periods of high temperature and radiation load, thereby reducing its susceptibility to damage [28,29].

During the winter-spring months, *P*. *boldus* experienced a drastic decrease in PI_ABS_ (Figure 4), a parameter that summarizes light trapping, trapped exciton flux, and electron transfer [30,31,32]. Mild temperatures during winter also limit plant growth [33,34], but *P*. *boldus* seems particularly sensitive to mild temperatures in both winter and spring. The deviation of the average PI_ABS_ value was negative in *P*. *boldus* during winter and spring (−∆PI_ABS_ 50 and 38%, respectively) but positive in *C*. *odorifera* in the same seasons (+∆PI_ABS_ 52 and 37%, respectively). Reductions in photosynthetic capacity (i.e., F_V_/F_M_) during winter have also been reported in Mediterranean species [14,26,35], and were associated with damage in the photosynthetic apparatus. In addition, the winter photoinactivation degree of *P*. *boldus* (PhI = 0.35) was higher than that reported in the Mediterranean *N. oleander* and *Myrtus communis* L. (PhI = 0.27 and 0.24, respectively [15]), corroborating its higher sensitivity and inactivation of PSII during winter.

The low photosynthetic efficiency of *P*. *boldus* was corroborated by the highest F_0_ (basic fluorescence) and ABS/RC (apparent antenna size of an active PSII) throughout the year. The higher F_0_ indicates destruction of PSII reaction centers and physical separation of the PSII from associated pigment antennae [36], whereas relatively larger PSII antenna size suggests the presence of a non-QA reducing reaction center [31], which occurs when overexcitation of the photosynthetic apparatus of *P*. *boldus* cannot appropriately be transferred to photosynthetic electron transport [5]. These results corroborate the sustained decreases in PSII efficiency of *P*. *boldus* under field conditions and its inability to balance photodamage and repair. It is known that *P*. *boldus* exerts strict stomatal control under stressful conditions [37], which adds a ‘bottleneck’ of electrons and contributes to its decreased photochemistry capacity. Thus, in general, *C*. *odorifera* showed a higher utilization of the absorbed energy in electron transport and, therefore, higher CO_2_ assimilation than *P*. *boldus*.

## 4. Materials and Methods

### 4.1. Characteristics of the Study Site and Plant Material

The experimental site was in the Las Brisas Experimental Station (35°34′ S, 72°06′ W) of the Universidad de Chile, San Javier, Chile. The site is located on a north-facing slope and is characterized by a Mediterranean-type climate with an annual average temperature of 14.2 °C and precipitation of 816 mm (70% concentrated during the winter months) and scant precipitation in summer. The average minimum and maximum temperatures are 5.0 and 29.5 °C, and the dry period is around 7 months [38]. Summer is typically hot and dry, with a maximum daily temperature of 36 °C during January. The soil type is sandy clay (47% sand, 17% lime, 36% clay) with a pH of 6.1 and low fertility and belongs to the Treguaco soil association (Dystric Xerochrepts, according to Soil Taxonomy classification). The electrical conductivity is 0.03 dS m^−1^ and the organic matter content is 1.5%. Available N, P, and K are 4, 8, and 168 mg kg^−1^, respectively. The soil profile is deep and well-developed, formed from metamorphic rocks, especially highly weathered mica schists and gneiss, with brown colors in 10YR and 7.5YR hues on the surface, transitioning to dark brown in the 7.5YR hue at depth [39]. The Treguaco soil predominantly occupies the high sectors and the eastern slope and is well-structured and friable, with good porosity that allows for good root development [39]. To characterize the climatic information during the study period, we obtained data from a weather station located 15 km from the planting site (Estancia Flora weather station), which belongs to the Instituto de Investigaciones Agropecuarias (https://agrometeorologia.cl accessed on 25 November 2025). From this station, we obtained average monthly values for temperature (minimum and maximum, °C), cumulated precipitation (mm), and total solar radiation (Mj m^−2^) from August 2024 to September 2025.

In 2019, seeds from *P*. *boldus* and *C*. *odorifera* were collected in a site located 20 km from the study area and cultivated in a nursery for 9 months. The plantation was carried out in July 2020, at a planting density of 1 × 1 m. The mean seedling height for *P*. *boldus* and *C*. *odorifera* was 15.4 and 33.8 cm, respectively. The experiment followed a randomized complete block design with five replicates, which were located continuously in the study area. A rectangular plot of 10 seedlings in a 2 × 5 m seedling arrangement (spacing of 1 × 1 m) was the experimental plot. Each replicate had 20 seedlings, and a total of 100 seedlings were planted (2 species × 5 replicates × 10 seedlings per replicate = 100 seedlings). During summer 2020–2021, seedlings were irrigated with 2 L^−1^ week^−1^ during five months (i.e., November 2020 to March 2021). After that, no more irrigations were applied. The present study was performed using five-year-old seedlings, where the recorded survival was 81% and 33% for *C*. *odorifera* and *P*. *boldus*, respectively.

### 4.2. Seasonal Variations in Chlorophyll Fluorescence

From the surviving seedlings, we took three individuals per species in 3 replicates chosen randomly (i.e., a total of 18 plants from replicates 1, 2, and 5) and used them for the subsequent measurements of chlorophyll fluorescence. Measurements were carried out between the 8th and 13th day of each month, from September 2024 to September 2025. One healthy and attached leaf of each seedling was marked for chlorophyll fluorescence measurements, which was assessed using an OS30p+ fluorometer (OptiSciences, Hudson, NH, USA). Leaves were dark-adapted for 30 min using leaf clips, and then, the OJIP kinetics of transients were induced with a pulse of saturating red light of 3500 µmol (photon) m^−2^ s^−1^. This saturating pulse was chosen based on previous tests to avoid over-reduction in the photosynthetic apparatus [5]. The fluorescence intensity was recorded from 20 µs to 3 s, and the data were analyzed using the JIP-test [30]. The introduced basic fluorescence parameters are listed in Table 2.

### 4.3. Data Analyses

For the analysis of chlorophyll fluorescence, the monthly data were grouped in seasons of the year for the Southern Hemisphere (i.e., summer: December, January, and February; autumn: March, April, and May; winter: June, July, and August; and spring: September, October, and November). A principal component analysis (PCA) with the Equamax rotation of the axis was first carried out with the aim of exploring the relationship between species and photosynthetic performance. This analysis was performed in InfoStat version 2020 (Group Infostat, Universidad Nacional de Córdoba, Argentina). Data were standardized and the 13 physiological variables were included as ‘response variables’, whereas the factor species (*C*. *odorifera* and *P*. *boldus*) was included as a ‘classification variable’. After that, we evaluated the differences among species, seasons, and the interaction between factors through two-way analysis of variance. The model used was

(1) Y = μ + R + Species + Season + Species × Season + ε

where Y is the observed phenotypic value, µ is the overall mean, R is the random effect of replicates, species is the fixed effect of species (*C*. *odorifera* and *P*. *boldus*), season is the fixed effect of a season of the year (summer, autumn, winter, and spring), Species × Season is the interaction between both factors, and ε represents the experimental random error. Significant value means were separated by Tukey’s test for *p* < 0.05. To meet the assumptions of normality and constant variances, traits were transformed when needed. Additionally, we estimated the winter photoinactivation degree (PhI) for Mediterranean evergreen woody plants as (PhI = 1 − (F_V_/F_Mwinter_/F_V_/F_Mspring_) [15]. We calculated the average PI_ABS_ with all data in each season (value 0 in Figure 4) and then calculated the percentage of deviation from this average for each species in each season separately. This was indicated as +∆PI_ABS_ (positive deviation) and −∆PI_ABS_ (negative deviation) in Figure 4. This statistical analysis was performed with SPSS version 22.0 software (SPSS Inc., Chicago, IL, USA).

## 5. Conclusions

*C*. *odorifera* had superior photosynthetic performance compared to *P*. *boldus* across the different seasons of the year. This species showed a higher utilization of the absorbed energy in electron transport and, therefore, higher photosynthetic performance, which is consistent with its occurrence on north-facing slopes with higher temperatures and radiation loads. In contrast, *P*. *boldus* was considerably depressed during the entire year. The higher reduction in photochemical efficiency of *P*. *boldus* during the winter–spring months (i.e., −ΔPI_ABS_) suggests that the mildly low temperatures of Mediterranean-type ecosystems may also be a considerable stress factor for this species, as it limits the photon trapping and photochemical phase of photosynthesis. As PSII function in *P*. *boldus* is more sensitive to environmental conditions in the early stages of establishment, the selection of this species for the restoration of Mediterranean-type climate sites must include silvicultural treatments aimed at improving survival and growth conditions across the entire year (e.g., higher and more frequent irrigation doses in summer and protection from low temperatures in winter–spring).

## Figures and Tables

**Table 1:** F-values and significance from the analysis of variance on Chl a fluorescence parameters in *P*. *boldus* and *C*. *odorifera* during the 2024–2025 season.

Parameter	Effect		
	Species	Season	Species × Season
V_K_	0.4 ns	0.9 ns	1.4 ns
ABS/RC	7.5 **	1.1 ns	0.4 ns
F_0_	22.3 **	3.1 *	0.2 ns
F_M_	2.8 ns	8.7 **	0.3 ns
F_V_/F_M_	55.1 **	4.3 **	3.2 *
F_V_/F_0_	97.7 **	8.5 **	3.1 *
ΔV_IP_	54.6 **	1.6 ns	0.6 ns
δR_0_	42.3 **	7.7 **	1.9 ns
Sm	0.6 ns	6.2 **	0.3 ns
ψE_0_	5.0 *	3.3 *	1.8 ns
ψR_0_	52.0 **	1.7 ns	0.5 ns
PI_ABS_	29.9 **	18.1 **	6.0 **
PI_TOT_	53.7 **	10.2 **	4.8 **

** = *p* < 0.001; * = *p* < 0.05; ns = non-significant (*p* > 0.05). F0 = minimal fluorescence from a dark-adapted leaf; FM = maximal fluorescence from a dark-adapted leaf; FV/FM = maximum quantum yield of primary PSII photochemistry; FV/F0 = ratio between variable and minimal fluorescence; Sm = normalized area above the curve from F0 to FM; ABS/RC = effective antenna size of an active reaction center (RC); PIABS = performance index for energy conservation from photons absorbed by PSII to the reduction of intersystem electron acceptors; PITOT = performance index for energy conservation from photons absorbed by PSII antenna to the reduction of PSI acceptors; ψE0 = probability that the energy of a trapped excitation is used for electron transport beyond QA; ψR0 = quantum yield for reduction in the end electron acceptors at the PSI acceptor side; δR0 = efficiency with which an electron from the intersystem electron carriers is transferred to reduce end electron acceptors at the PSI acceptor side; VK = relative fluorescence at the K-step; ΔVIP = efficiency with which a PSII trapped electron is transferred to final PSI acceptors.

**Table 2:** Main terms obtained for the analysis of the fluorescence transient OJIP.

**Technical fluorescence parameters**	
F_0_	Minimal fluorescence from a dark-adapted leaf
F_M_	Maximal fluorescence from a dark-adapted leaf
F_V_	Maximal variable fluorescence from a dark-adapted leaf
F_V_/F_M_	Maximum quantum yield of primary PSII photochemistry
F_V_/F_0_	Ratio between variable and minimal fluorescence
O step	Origin fluorescence value at 20 µs
K step	Fluorescence value at 0.3 ms
J step	Fluorescence value at 2 ms
I step	Fluorescence value at 30 ms
P step	Maximum fluorescence or F_M_
**JIP-test derived parameters**	
Sm	Normalized area above the curve from F_0_ to F_M_
ABS/RC	Effective antenna size of an active reaction center (RC)
PI_ABS_	Performance index for energy conservation from photons absorbed by PSII to the reduction of intersystem electron acceptors
PI_TOT_	Performance index for energy conservation from photons absorbed by PSII antenna to the reduction of PSI acceptors.
ψE_0_	Probability that the energy of a trapped excitation is used for electron transport beyond QA
ψR_0_	Quantum yield for reduction in the end electron acceptors at the PSI acceptor side
δR_0_	Efficiency with which an electron from the intersystem electron carriers is transferred to reduce end electron acceptors at the PSI acceptor side
V_K_	Relative fluorescence at the K-step
ΔV_IP_	Relative contribution of electron flow to the PSI end acceptors (i.e., ferredoxin and NADPH)

PI_TOT_ was obtained as PI_ABS_ × δR_0_/(1 − δR_0_), whereas δR_0_ was obtained as ψR_0_/ψE_0_ [20].

## Data Availability

The raw data supporting the conclusions of this article will be made available by the authors on request. The data are not publicly available due to privacy.

## Abbreviations

The following abbreviations are used in this manuscript:

F_0_	Minimal fluorescence from a dark-adapted leaf
F_M_	Maximal fluorescence from a dark-adapted leaf
F_V_	Maximal variable fluorescence from a dark-adapted leaf
F_V_/F_M_	Maximum quantum yield of primary PSII photochemistry
F_V_/F_0_	Ratio between variable and minimal fluorescence
O step	Origin fluorescence value at 20 µs
K step	Fluorescence value at 0.3 ms
J step	Fluorescence value at 2 ms
I step	Fluorescence value at 30 ms
P step	Maximum fluorescence or F_M_
Sm	Normalized area above the curve from F_0_ to F_M_
ABS/RC	Effective antenna size of an active reaction center (RC)
PI_ABS_	Performance index for energy conservation from photons absorbed by PSII to the reduction of intersystem electron acceptors
PI_TOT_	Performance index for energy conservation from photons absorbed by PSII antenna to the reduction of PSI acceptors.
ψE_0_	Probability that the energy of a trapped excitation is used for electron transport beyond QA
ψR_0_	Quantum yield for reduction in the end electron acceptors at the PSI acceptor side
δR_0_	Efficiency with which an electron from the intersystem electron carriers is transferred to reduce end electron acceptors at the PSI acceptor side
V_K_	Relative fluorescence at the K-step
ΔV_IP_	Relative contribution of electron flow to the PSI end acceptors (i.e., ferredoxin and NADPH)
